# A possible origin population of pathogenic intestinal nematodes, *Strongyloides stercoralis*, unveiled by molecular phylogeny

**DOI:** 10.1038/s41598-017-05049-x

**Published:** 2017-07-07

**Authors:** Eiji Nagayasu, Myo Pa Pa Thet Hnin Htwe Aung, Thanaporn Hortiwakul, Akina Hino, Teruhisa Tanaka, Miwa Higashiarakawa, Alex Olia, Tomoyo Taniguchi, Soe Moe Thu Win, Isao Ohashi, Emmanuel Igwaro Odongo-Aginya, Khin Myo Aye, Mon Mon, Kyu Kyu Win, Kei Ota, Yukari Torisu, Siripen Panthuwong, Eisaku Kimura, Nirianne M. Q. Palacpac, Taisei Kikuchi, Tetsuo Hirata, Shidow Torisu, Hajime Hisaeda, Toshihiro Horii, Jiro Fujita, Wah Win Htike, Haruhiko Maruyama

**Affiliations:** 1 Division of Parasitology, Department of Infectious Diseases, Faculty of Medicine, University of Miyazaki, Miyazaki, Japan; 2 Department of Microbiology, University of Medicine 1, Yangon, Republic of the Union of Myanmar; 3 Department of Internal Medicine, Faculty of Medicine, Prince of Songkla University, Hat Yai, Thailand; 4 Department of Environmental Parasitology, Tokyo Medical and Dental University, Tokyo, Japan; 5 Department of Endoscopy, University of the Ryukyus Hospital, Okinawa, Japan; 6 Department of Infectious, Respiratory, and Digestive Medicine, Graduate School of Medicine, University of the Ryukyus, Okinawa, Japan; 7 Department of Microbiology and Immunology, Faculty of Medicine, Gulu University, Gulu, Uganda; 8 Department of Parasitology, Graduate School of Medicine, Gunma University, Maebashi, Gunma, Japan; 9 Department of Medical Technology, Koshimizu Red Cross Hospital, Koshimizu, Japan; 10 Department of Emergency and General Medicine, Koshimizu Red Cross Hospital, Koshimizu, Japan; 11 Section of Oncopathology and Regenerative Biology, Faculty of Medicine, University of Miyazaki, Miyazaki, Japan; 12 Department of Molecular Protozoology, Research Institute for Microbial Diseases, Osaka University, Suita, Osaka Japan; 13 Veterinary Teaching Hospital, Faculty of Agriculture, University of Miyazaki, Miyazaki, Japan

## Abstract

Humans and dogs are the two major hosts of *Strongyloides stercoralis*, an intestinal parasitic nematode. To better understand the phylogenetic relationships among *S*. *stercoralis* isolates infecting humans and dogs and to assess the zoonotic potential of this parasite, we analyzed mitochondrial Cox1, nuclear 18S rDNA, 28S rDNA, and a major sperm protein domain-containing protein genes. Overall, our analyses indicated the presence of two distinct lineages of *S*. *stercoralis* (referred to as type A and type B). While type A parasites were isolated both from humans and dogs in different countries, type B parasites were found exclusively in dogs, indicating that the type B has not adapted to infect humans. These epidemiological data, together with the close phylogenetic relationship of *S*. *stercoralis* with *S*. *procyonis*, a *Strongyloides* parasite of raccoons, possibly indicates that *S*. *stercoralis* originally evolved as a canid parasite, and later spread into humans. The inability to infect humans might be an ancestral character of this species and the type B might be surmised to be an origin population from which human-infecting strains are derived.

## Introduction

The genus *Strongyloides* comprises more than 50 obligate gastrointestinal parasitic nematode species that infect wild animal including birds, reptiles, amphibians, and mammals, as well as humans, livestock, and companion animals^1, 2^. In humans, two species are known to cause infection: *S*. *stercoralis* (Bavay 1876) and *S*. *fuelleborni* (consisting of two subspecies, *S*. *fuelleborni fuelleborni* (Von Linstow 1905) and *S*. *fuelleborni kellyi* (Viney 1991). While *S*. *stercoralis* has a cosmopolitan distribution, mainly in tropical and sub-tropical regions, human infection with *S*. *fuelleborni fuelleborni* only occurs sporadically in Africa. *S*. *fuelleborni kellyi* was once considered a subspecies of *S*. *fuelleborni fuelleborni* based on morphological resemblances^3^ together with results of an isoenzyme electrophoretic study^4^, although more recent molecular phylogenetic analyses do not support this subspecies relationship^5^. So far, human infections with *S*. *fuelleborni kellyi* have been reported only in Papua New Guinea^6^.

In dogs, *S*. *stercoralis* and *S*. *planiceps* are two major species known to cause infections. The first attempt to distinguish human and dog isolates of *S*. *stercoralis* by molecular analysis was carried out by Ramachandran *et al*.^7^ using PCR-RFLP on the 28S rDNA locus^7^. The results showed different RFLP patterns between the two host isolates, although only four human and one dog isolate were used. Later, Hasegawa *et al*.^8^ reported a polymorphism in the 18S rDNA gene between two human and five dog isolates of *S*. *stercoralis* in comparison with other *Strongyloides* spp^8^. The polymorphism is in the HVR-I region, one of four “hyper-variable” regions identified in the 18S rDNA locus^8^. (Note: Hasegawa’s nomenclature to designate hyper-variable regions on the *S*. *stercoralis* 18S rDNA gene differs from the more widely used system to mark hyper-variable regions (V1 to V9) on eukaryote 18S rDNA genes)^9^. The nature of the HVR-I polymorphism observed between isolates of *S*. *stercoralis* is a single base insertion/deletion (indel) that results in a stretch of either four or five thymidine nucleotides within the HVR-I region (4T or 5T alleles). In their study, all dog isolates possessed the 4T/4T genotype, while two human isolates displayed the 5T/5T genotype. Recently, Hasegawa *et al*.^10^ also showed that *S*. *stercoralis* isolates from humans, chimpanzees, and dogs can be grouped into dog-parasitic and primate-parasitic clades using the mitochondrial Cox1 gene as a genetic marker from human (n = 5), chimpanzee (n = 2), and dog isolates (n = 2)^10^. The first report to determine worm genotypes at a population level were conducted in Cambodia by Schar *et al*.^11^, in which 18S rDNA was used as a marker^11^. Interestingly, 4T/4T genotypes were found in seven out of 29 human hosts (dogs were not included in the study). Thus, although lines of evidence suggest that some degree of genetic differentiation between human and dog isolates already exists, the usefulness of the HVR-I polymorphism to distinguish human and dog strains still needs verification. From a public health point of view, it is important to know the degree of cross-transmission of dog-adapted isolates to humans.

To gain a better understanding of the phylogenetic relationships among *S*. *stercoralis* isolates infecting humans and dogs, and to assess their zoonotic potential, we collected *S*. *stercoralis* samples mainly from Myanmar, where strongyloidiasis is endemic in both humans and dogs. We analyzed mitochondrial Cox1, nuclear 18S rDNA, 28S rDNA, and the major sperm protein domain-containing protein gene (MSP gene, hereafter), and found that *S*. *stercoralis* consists of two genetically distinct lineages.

## Results

### Cox1 haplotypes

Cox1 sequences from 571 *Strongyloides* worms isolated from 159 hosts were collapsed into 100 haplotypes. The average pairwise distances of nucleotide sequences (711 bp) and translated amino acid sequences (237 positions) among the haplotypes were 0.036 (range, 0.001–0.079) and 0.003 (range, 0–0.025), respectively (Supplementary Tables S1 and S2).

**Figure 1:**
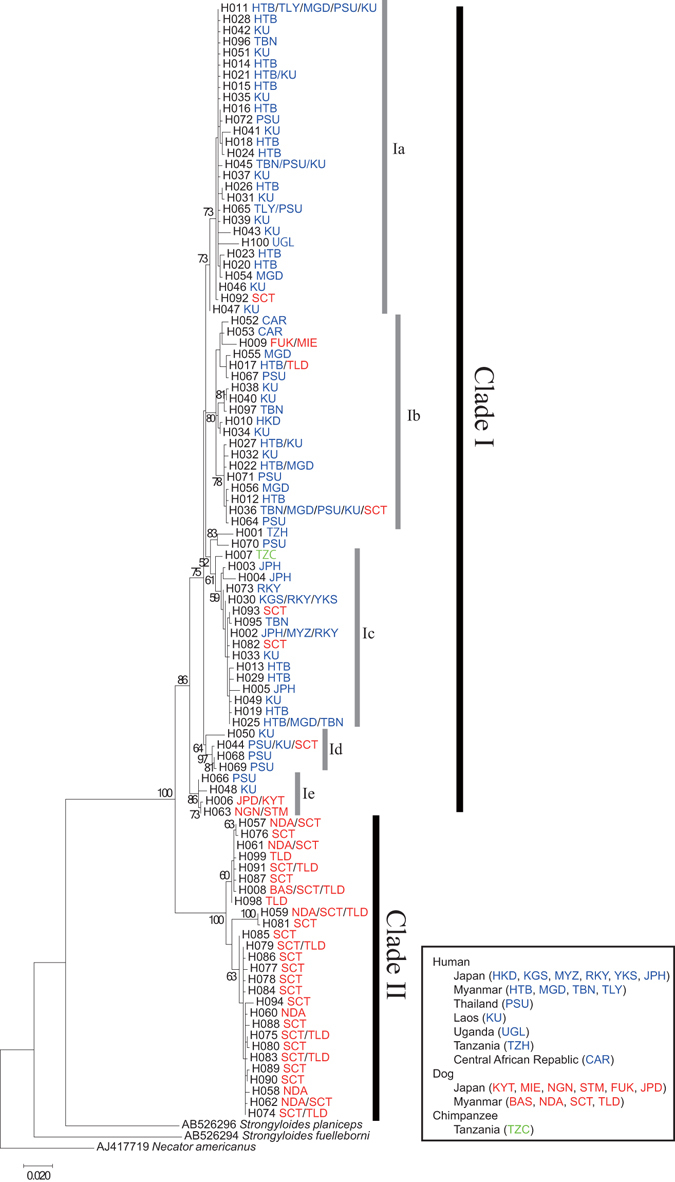
Maximum-likelihood tree built for *Strongyloides* isolates using a portion of the mitochondrial Cox1 gene. MEGA7 software was used to build the tree using a dataset of Cox1 (710 bp) gene haplotypes based on the Tamura-Nei model. Two/Three-letter codes next to the haplotype number indicate haplotypes from the respective hosts. Numbers at nodes indicate percent bootstrap values generated from 500 replicates. Scale bar denotes 0.02 changes per nucleotide site.

A maximum-likelihood tree was constructed to assess the phylogenetic relationships between these haplotypes (Fig. 1). Two distinct groups were observed. The first group (clade I) consisted of all human-derived worms and some dog-derived worms. In contrast, the second group (clade II) was composed solely of dog-derived worms collected in Myanmar. Subdivisions among clade I haplotypes were also observed (clade Ia–e). Clade Ia was mostly composed of Southeast Asian human isolates (Myanmar, Thailand, and Laos) together with one Myanmar dog-derived isolate and Ugandan human-derived isolate. Clade Ib was composed of human-derived isolates from 5 countries (Myanmar, Thailand, Laos, Japan, and Central Africa) and dog isolates from Myanmar and Japan. Clade Ic was composed of human isolates from Myanmar, Laos, and Japan, and dog isolates from Myanmar. It contained all of the Japanese human isolates except for one isolate from Hokkaido (HKD) that was grouped into clade Ib (H010). It is of note that clade Ic was supported by a relatively weak bootstrap value (61%). Clade Id was a relatively small clade containing Southeast Asian human-derived (Thailand and Laos) and Myanmar dog-derived isolates. Clade Ie was also a small group containing two Japanese dog-derived haplotypes and human-derived haplotypes from Laos and Thailand.

**Figure 2:**
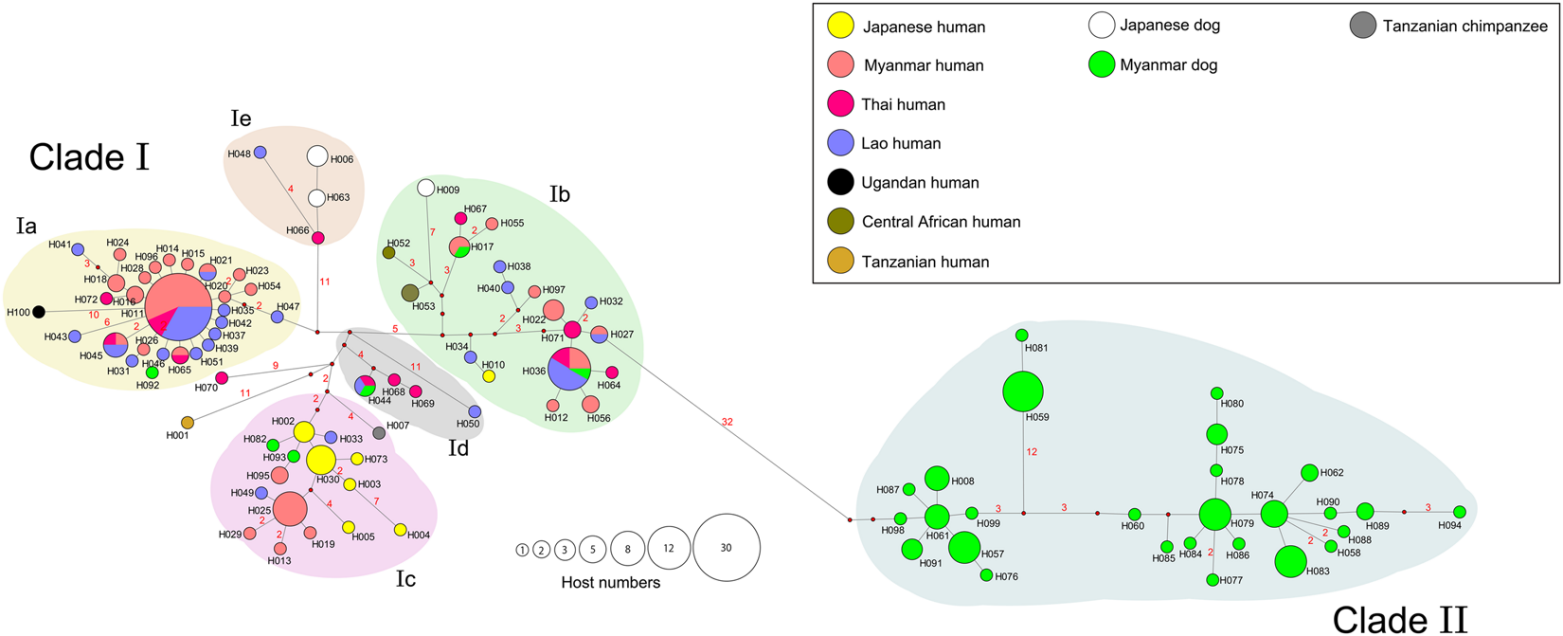
Median-joining haplotype network for the mitochondrial Cox1 gene. Each circle represents one haplotype. The size of the circle represents the number of hosts that harbor the given haplotype. The color inside the circle indicates the host species/geographical origin (country). Numbers beside the branches indicate the number of mutational steps between haplotypes (no number is shown in case of a single-step difference). Branch lengths are roughly proportional to the number of mutational steps.

The same dataset was used to construct a Median-joining phylogenetic network (Fig. 2). In the case of clade Ia, a core haplotype (H011) was surrounded by other haplotypes. H011 was the most prevalent haplotype in both Myanmar and Laos. In other clades, less prominent core haplotypes were observed that included H036, H025, H061, H074, and H079. Of 116 worms from 36 dog hosts, 38 worms from 11 dog hosts possessed Cox1 haplotypes belonging to clade I (Figs 1 and 2). These 38 worms were typed to have one of the following nine Cox1 haplotypes (clade Ia: H092; clade Ib: H009, H017, H036; clade Ic: H082, H093; clade Id: H044; clade Ie: H006, H063). H017, H036, and H044 were shared by human isolates. H092, H082, H093, H063, and H006 were distinctively found in dogs. The difference from the closest haplotypes found in human isolates was minimal, with only one or two nucleotide changes (Fig. 2). Nevertheless, 33 out of 34 Cox1-clade I worms from canine hosts had the 4T/4T 18S HVR-I genotype (Table 1), indicating these isolates may not be human-adapted (described below).

**Table 1:** 18S rDNA hyper-variable region I (HVR-I) genotypes of *Strongyloides* worms isolated from humans and dogs.

Cox 1 clade	HVR-I genotype	Human isolates	Dog isolates
I	5T/5T	351 (74)	1 (1)
	4T/5T	9 (4)	0
	4T/4T	10 (7)	33 (9)
II	5T/5T	0	0
	4T/5T	0	0
	4T/4T	0	67 (28)
		370 (85)	101 (38)

The number of worms (outside the brackets) with respective genotypes are shown. Numbers inside the brackets indicate the number of hosts from which worms with respective genotypes were isolated.

### 18S rDNA 458T/A polymorphism and HVR-I genotypes

Within the 333-bp region that we used for nematode species identification, one polymorphic site was identified at position 458 of the reference (AF279916). This is the same polymorphism reported in a *S*. *stercoralis* population from humans in Cambodia^11^. The genotypes of this polymorphism were determined for all 521 samples that we collected. Among them, virtually all Myanmar and Thai worms possessed genotype T/T, regardless of the host (Table 2), with five exceptions. The five worms were isolated from three HIV-positive patients recruited at Mingaladon Specialist Hospital (MGD) with CD4-positive T lymphocyte counts of 10, 27, and 52 cells/μL. All Japanese isolates carried genotype A/A regardless of the host species, with one exception. That one isolate was from a human host in Hokkaido (HKD) and had a heterozygous genotype (T/A).

**Table 2:** Genotypes of 18S rDNA 458T/A polymorphism

Country	Host species	Location (host #)	Genotype		
			T/T	T/A	A/A
Myanmar	Human	HTB (24)	119	0	0
		TBN (6)	27	0	0
		TLY (6)	28	0	0
		MGD (13)	55	0	5
	Dog	BAS (1)	1	0	0
		NDA (3)	8	0	0
		SCT (22)	62	0	0
		TLD (5)	13	0	0
Thailand	Human	PSU (16)	106	0	0
Japan	Human	MYZ (1)	0	0	5
		KGS (1)	0	0	7
		RKY (6)	0	0	34
		YKS (1)	0	0	8
		HKD (1)	0	1	2
	Dog	KYT (1)	0	0	11
		MIE (1)	0	0	5
		NGN (1)	0	0	10
		STM (1)	0	0	3
		FUK (1)	0	0	3
Uganda	Human	UGL (1)	8	0	0

Genotypes for the polymorphism at position 458 of the reference sequence (AF279916; NCBI nucleotide database) were determined for *S*. *stercoralis* samples collected in the study.

A single-base insertion/deletion that results in either a stretch of four or five thymidine nucleotides within the 18S rDNA HVR-I gives rise to ‘4T’ or ‘5T’ alleles. The HVR-I genotypes were determined for 471 out of 521 samples (370 human isolates and 101 dog isolates). In humans, 94.5% (351 out of 370) of the isolated *Strongyloides* worms bore the HVR-I genotype 5T/5T. In stark contrast, virtually all (99.0%, 100 out of 101) worms from dogs carried genotype 4T/4T (Table 1). The minor 4T/5T and 4T/4T genotypes were found in four and seven humans, respectively. In this calculation, the host number was counted twice if a single host produced *S*. *stercoralis* with two different genotypes. Thus, the actual number of humans who were the hosts of either genotype 4T/5T or 4T/4T was eight (not the sum of four and seven). For these eight human hosts, we looked at the genotypes of individual worms from the same host (Table 3). We noticed that worm populations from these human hosts were always mixtures of either 5T/5T + 4T/5T, 5T/5T + 4T/4T, or 5T/5T + 4T/5T + 4T/4T. We did not see any human case where only 4T/4T genotypes were found. In six of the eight subjects (Table 3), all worms from the same host shared the same Cox1 haplotype, even if their HVR-I genotypes differed.

**Table 3:** 18S rDNA hyper-variable region I (HVR-I) genotypes of individual *Strongyloides* worms from selected human hosts.

	Individual worm#						
	1	2	3	4	5	6	7
HKD001	5T/5T	4T/5T					
(Japan)	10	10					
HTB010	5T/5T	4T/5T	4T/5T	4T/5T	4T/5T	4T/5T	4T/4T
(Myanmar)	13	13	13	13	13	13	13
HTB065	5T/5T	4T/5T	4T/5T	4T/4T			
(Myanmar)	11	11	11	11			
HTB169	5T/5T	5T/5T	5T/5T	5T/5T	4T/4T		
(Myanmar)	27	27	27	27	27		
MGD053	5T/5T	5T/5T	5T/5T	5T/5T	4T/4T		
(Myanmar)	22	22	22	22	36		
PSU031	4T/5T	4T/4T	4T/4T	4T/4T			
(Thailand)	36	36	36	36			
PSU033	5T/5T	5T/5T	5T/5T	5T/5T	5T/5T	4T/4T	
(Thailand)	69	68	68	68	68	68	
PSU034	5T/5T	5T/5T	4T/4T				
(Thailand)	70	70	70				

Eight human hosts were selected based on the presence of at least one isolated *Strongyloides* worm with a rare HVR-I genotype (4T/4T or 4T/5T). Two to seven worms/host were successfully genotyped. Each box represents an individual worm. The upper row shows the HVR-I genotype, and the lower row contains the Cox1 haplotype number.

### 28S rDNA haplotypes

**Figure 3:**
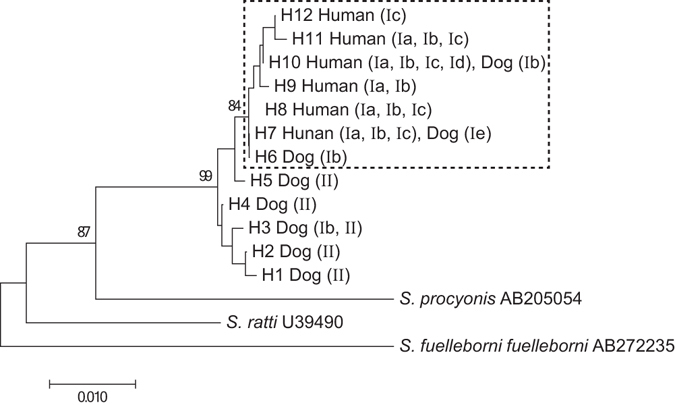
Neighbor-joining tree built for *Strongyloides* isolates using a portion of the nuclear 28S rDNA gene. MEGA7 software was used to build the tree using a dataset of 28S rDNA (743 nucleotide positions) gene haplotypes based on the Tamura-Nei model. A square depicted by a dashed line indicates a distinct clade, composed of haplotypes H6–H12. Letters inside the brackets indicate the Cox1 clade to which each isolate belongs with respect to the 28S rDNA haplotype of each sample.

A portion of the 28S rDNA gene (733–736 bp in length, depending on the isolate) was successfully obtained from 311 isolates from 86 hosts. Twelve haplotypes were identified. The phylogenetic relationships between haplotypes are shown in Fig. 3. One distinct clade (bootstrap value, 84%) comprising H6–H12 was formed. All human isolates belonged to this clade, and all isolates typed as Cox1 clade I belonged to this clade, with one exception (Table 4). This exception was an isolate from a Myanmar dog, and was typed as Cox1 clade Ib and 28S rDNA haplotype H3. Haplotype H10 was the most predominant haplotype, accounting for 60.5% (188 out of 311) of the all isolates (Table 5). This haplotype was not only the most prevalent among humans in Myanmar and Thailand, but also the only haplotype found in all four countries studied (Myanmar, Thailand, Japan, and Uganda).

**Table 4:** 28S rDNA haplotypes identified in humans and dogs.

Cox 1 clade	28S rDNA haplotype	Human isolates	Dog isolates
I	H6, H7, H8, H9, H10, H11, H12	265 (68)	29 (6)
	H1, H2, H3, H4, H5	0	1 (1)
II	H6, H7, H8, H9, H10, H11, H12	0	0 (0)
	H1, H2, H3, H4, H5	0	16 (11)
		265 (68)	46 (18)

28S haplotypes were grouped into 2 categories (H1–H5 or H6–H12) based on the phylogenetic analysis (Fig. 3). Numbers outside the brackets indicate the number of nematodes that belong to each category. Numbers inside the brackets indicate the number of hosts from which worms belonging to the respective categories were isolated.

**Table 5:** 28S rDNA haplotypes identified in *S*. *stercoralis* isolated from humans or dogs.

Location and host	28S rDNA haplotype											
	H1	H2	H3	H4	H5	H6	H7	H8	H9	H10	H11	H12
Myanmar dog	1 (1)	3 (3)	8 (7)	1 (1)	4 (4)	0	0	0	0	1 (1)	0	0
Japanese dog	0	0	0	0	0	7 (2)	21 (3)	0	0	0	0	0
Myanmar human	0	0	0	0	0	0	23 (7)	5 (2)	8 (2)	116 (41)	3 (2)	0
Thai human	0	0	0	0	0	0	1 (1)	5 (2)	13 (2)	59 (10)	0	0
Japanese human	0	0	0	0	0	0	0	0	0	9 (4)	17 (6)	3 (1)
Ugandan human	0	0	0	0	0	0	0	0	0	3 (1)	0	0

Numbers outside the brackets indicate the number of isolates typed as respective haplotypes. Numbers inside the brackets indicate the number of host animals from which worms with respective haplotypes were isolated. Note that the sum of the host numbers (103) shown on this table is more than the actual host number (86), because some hosts harbored worms belonging to different haplotypes.

### MSP gene haplotypes

The nuclear-protein–coding gene MSP, has been used in nematode molecular systematics studies^12^. Our preliminary database search of the *S*. *stercoralis* genome identified at least three MSP genes, SSTP_0000560400.1, SSTP_0000955200.1, and SSTP_0000122000.1, with predicted gene products of 127, 127, and 575 amino acids, respectively. Although these reference sequences had not been registered in the NCBI protein database at the time of this writing, they were available through the WormBase Parasite (parasite.wormbase.org). The first two genes showed a high level of homology with each other at the nucleotide level (91% identical), and were not used in the present study to avoid potential problems associated with analyzing paralogous sequences. As a result, we chose SSTP_0000122000.1 for our study.

**Figure 4:**
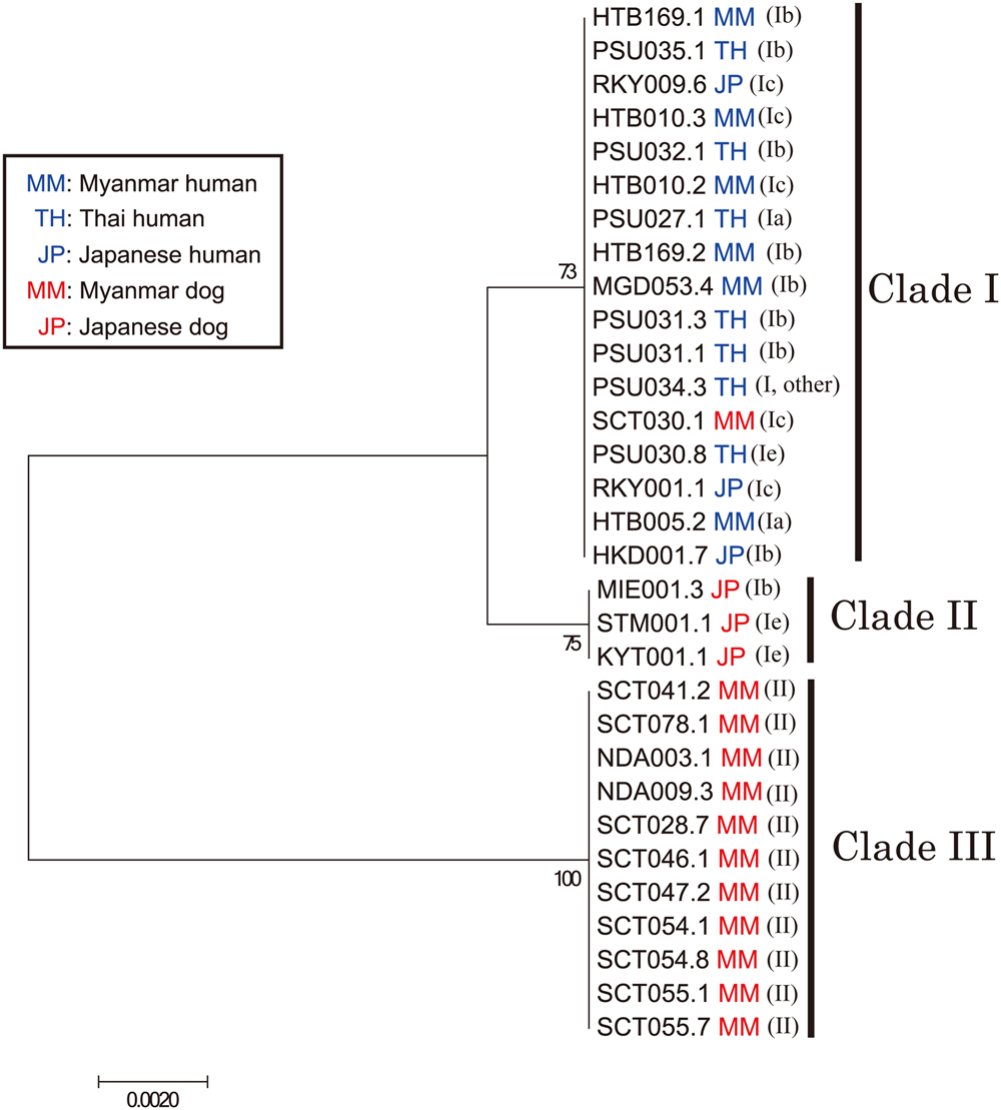
Maximum-likelihood tree built for selected *Strongyloides* isolates using a portion of a nuclear major sperm protein domain-containing protein (MSP) gene. MEGA7 software was used to build the tree using a dataset of MSP gene (551 bp) based on the Tamura-Nei model. Two-letter codes next to the isolate names indicate types of hosts from which those were isolated. Letters inside the brackets indicate the Cox1 clade to which each isolate belonged.

We selected 31 samples (16 human-derived and 15 dog-derived), for phylogenetic analysis of the MSP gene. The sample selection was made arbitrarily to make the selected samples represent the observed diversity of the Cox1 haplotypes described above. Base on the maximum-likelihood tree, they were grouped into three clades (Fig. 4). All human isolates grouped as Cox1 clade I belonged to MSP clade I, while all dog isolates grouped as Cox1 clade II belonged to MSP clade III. Interestingly, all three isolates from Japanese dogs were grouped together as MSP clade II, although they belonged to different Cox1 subclades (either Ib or Ie).

### Morphometrics of the *S*. *stercoralis*isolates belonging to Cox1 clade II

The phylogenetic analyses using mitochondrial Cox1, nuclear 28S rDNA and MSP gene as markers suggested that *S*. *stercoralis* population isolated from dogs in Myanmar are genetically distinct. Because all the *S*. *stercoralis* Cox1 sequences deposited to NCBI database before the present study belonged to the Cox1 clade I, these clade II-worms appeared to represent a diverged member of this species that had not been recognized before. In order to know if this group of worms show any noticeable difference to the reported morphologies for this species, we conducted a morphometrics analysis using free-living male and female adult worms (Table 6). The worms were picked from an additional agar culture plate with fecal sample of a Myanmar dog.

**Table 6:** Morphometric analysis on *S*. *stercoralis* isolated from dog feces in Myanmar.

Free-living adult females			
	*S*. *stercoralis* (Cox1 clade II)	*S*. *stercoralis*	*S*. *procyonis*
	(Present study)	Little^13^	Little^13^
Number of examined specimens	17	31	24
Body length (mm)^*^	1.12 (1.02–1.22)	1.13 (0.92–1.7)	1.29 (1.0–1.6)
Body width (µm)^*^	61 (53–67)	62 (52–85)	65 (45–80)
Esophagus length (µm)^*^	145 (134–153)	145 (125–150)	170 (155–195)
% of body length^‡^	12.9 (0.08)	12.7 (0.22)	13.5 (0.21)
Mouth to vulva (µm)^*^	570 (517–638)	580 (470–820)	630 (540–750)
**Free-living adult males**			
Number of examined specimens	17	21	20
Body length (mm)^*^	0.97 (0.91–1.03)	0.89 (0.81–1.0)	0.97 (0.77–1.2)
Body width (µm)^*^	46 (42–54)	43 (40–50)	40 (30–50)
Body length/width ratio^†^	21.1	20.7^§^	24.3^§^
Esophagus length (µm)^*^	129 (121–142)	118 (110–125)	150 (130–170)
% of body length^‡^	13.4 (0.11)	13.4 (0.23)	15.6 (0.31)
Spicule length (µm)^*^	37 (35–39)	37 (35–40)	34 (30–37)
% of body length^‡^	3.9 (0.04)	4.2 (0.05)	3.5 (0.05)

The values are expressed as either actual number [not marked], mean (range) [marked with*], mean [marked with^†^] or mean (standard error) [marked with^‡^].

^§^These values were calculated by using the mean length and width data reported in Little^13^.

In case of free-living female adults (n = 17), the parameters measured (body length, body width, esophagus length, and mouth-to-vulva length) were almost identical to those reported by Little^13^. The relative length of esophagus to the total body length was more similar to *S*. *stercoralis* than to *S*. *procyonis*. As for the free-living male adults (n = 17), they were measured to be larger (9.0% longer and 7.0% wider) than the reported values for *S*. *stercoralis* on average. When values for the individual worms were looked at, they were within the ranges of reported values in most cases (15 out of 17 and 16 out of 17 worms for body length and body width measurements, respectively [data not shown]). The body length/width ratio, relative esophagus length, and absolute spicule length were nearly or completely identical to the reported values for *S*. *stercoralis*.

Cox1 haplotypes were determined for free-living male and female adults (n = 16 and n = 17, respectively) from the same culture plate. Among them, 24 worms belonged to the already identified clade II-haplotypes (H057, H059, H074, H078, H079, H083, and H091). The remaining 9 worms formed 7 additional haplotypes (H101–H107). A phylogenetic analysis using existing (H001–H100) and the newly-identified (H101–H107) haplotypes resulted in the positioning of H101–H107 to the clade II (data not shown). Hence, it was considered that either all of or predominant majority of the *S*. *stercoralis* population on this particular agar culture plate belong to Cox1 clade II, confirming that the morphometics data obtained above represent the morphology of the clade II worms.

## Discussion

We examined the phylogenetic relationships among *S*. *stercoralis* isolates from several human and dog populations using nuclear and mitochondrial markers. Overall, our analyses indicate two distinct lineages of *S*. *stercoralis*. We tentatively refer to them as *S*. *stercoralis* type A and type B. Cox1 clade I vs clade II haplotypes define *S*. *stercoralis* as type A or type B, respectively. The characteristics of both types are summarized in Table 7. *S*. *stercoralis* type A was found more ubiquitously than type B, being isolated from both human and dog hosts in multiple countries. So far, type B has only been isolated from dogs in Myanmar. All *S*. *stercoralis* Cox1 sequences recorded in the NCBI nucleotide database before this study belong to Cox1 clade I. Thus, we consider the type B worms to represent a diverged member of *S*. *stercoralis* that has not been recognized before.

**Table 7:** Tentative classification of *Strongyloides stercoralis* and their characteristics.

	*Strongyloides stercoralis*	
	Type A	Type B
Geographical distribution	Southeast Asia, Japan, Africa	Found only in Myanmar so far
Host	Human and dog	Found only in dogs so far
Cox1 clade	I	II
18S rDNA HVR-I allele	4T or 5T	4T
28S rDNA haplotype	3, 6, 7, 8, 9, 10, 11, and 12	1, 2, 3, 4, and 5
MSP gene clade	I, II	III

The morphological quantitative features of free-living male and female adult worms of Cox1 clade II were overall consistent with those reported for *S*. *stercoralis* by Little^13^ and differed much more from *S*. *procyonis*, the closest known *Strongyloides* species^14^. Considering that the morphological differences between *S*. *stercoralis* and *S*. *procyonis* are minimal, distinguishing between type A and type B *S*. *stercoralis* by light microscopy alone would be very difficult, although further verification is needed by direct comparison of Type A (Cox1 Clade I) worms using the same methodologies we employed for the morphometric analysis of Type B (Cox1 Clade II) worms.

18S rDNA HVR-I genotypes turned out to be a reasonably good indicator of the host species from which an isolate was derived. A great majority (351 out of 370, 94.5%) of human-derived isolates bore 18S rDNA hyper-variable region I (HVR-I) genotype 5T/5T, while virtually all (100 out of 101, 99.0%,) worms from dogs carried genotype 4T/4T. Based on this result, it appears that although experimental infection of human strains to dogs is reported^15^, this direction of cross-transmission appears to be quite rare in our study locations.

Some Cox1 clade I-worms isolated from dog had very similar (1–2 nucleotide difference) or identical Cox1 sequences to haplotypes found in human isolates. Nevertheless, these worms possessed a consistent HVR-I (4T/4T) genotype. These results indicate that the use of Cox1 sequences to differentiate human and dog strains belonging to clade I is not reliable. The presence of the 4T/4T HVR-I allele in isolates throughout the clade I (Ia–e) may indicate that the Cox1 diversity currently observed in clade I already existed before the divergence into human- and dog-adapted strains of *S*. *stercoralis* Type A.

HVR-I genotypes differ according to host species, suggesting that the divergence of dog and human strains might predate the dispersal of *S*. *stercoralis* to different geographical locations in Asia. On the other hand, the typing data for the 18S rDNA 458 T/A polymorphism show that they are different according to geographical location rather than to the host species. It might be possible that the nucleotide substitution at position 458 might have occurred independently in both dog- and human-infecting strains in Japan, without a true phylogenetic relationship. An alternative and more complex scenario is the double occurrence of divergence into dog and human strains, one in genotype T/T worms of the 458 T/A polymorphism and the other in genotype A/A worms. More typing data using samples from more diverse geographical locations with more genetic markers are required to draw any conclusions.

Eight human samples contained worms with at least one 4T allele (genotypes 4T/4T or 4T/5T). There was no case in which only the 4T/4T genotype was found in human samples (Table 3). These 4T/4T worms were always found together with 4T/5T or 5T/5T worms isolated from the same agar plate, indicating that a heterozygous parasitic female might have been the source of these worms. After sexual reproduction occurred on the agar-plate, these mixed-genotype progeny, including 4T/4T individuals, might have been produced. Regarding the means of reproduction of the free-living adults of *Strongyloides*, earlier cytological studies (Hammond and Robinson^16^ for *S*. *stercoralis* ^16^, Nigon and Roman^17^; Bolla and Robers^18^ for *S*. *ratti* ^17, 18^; Zaffagnini^19^; Triantaphyllou and Moncol^20^ for *S*. *papillosus* ^19, 20^, and Triantaphyllou and Moncol^20^ for *S*. *suis*)^20^ proposed sperm-dependent parthenogenesis (pseudogamy). This proposal was disproved later for *S*. *ratti* ^21^ and *S*. *pappilosus* ^22^ using molecular genetics approaches. In case of *S*. *stercoralis*, a recent report suggests that the sexual reproduction of free-living adults is highly likely to be occurring, on the basis of inbreeding coefficient estimation using genome-wide SNP data obtained from isolates from Myanmar humans^23^, although the direct demonstration is still awaited. At this point, inference was based solely on the observation that 4T/4T worms were always identified in the presence of 4T/5T and 5T/5T worms.

This study and a previous study in Cambodia^11^ identified a polymorphism at position 458 (numbering based on AF279916) in 18S rDNA. The majority of human and dog isolates in Myanmar possessed genotype T/T, while the opposite was true in isolates taken in Japan. Importantly, all three people who were infected with the rare A/A genotype in Myanmar were immunocompromised individuals with HIV infection. More study is needed to investigate the relationship between the worm genotypes and their potential as opportunistic pathogens. Alternatively, the apparent increase in this rare genotype among HIV-positive individuals might be the result of particular types of behaviors, such as prostitution for foreigners, that potentially increase the risk of acquiring both HIV infection and *S*. *stercoralis* of foreign origin. For the population of HIV-positive individuals enrolled this time, however, no data for their occupational histories are available. Therefore, future studies should carefully investigate both worm-genetic and host-behavioral factors.

Currently in Japan, virtually all strongyloidiasis cases occur in residents or former residents of the Okinawa prefecture or prefectures of the Kyushu island (southern regions of Japan)^24^. However, a rare strongyloidiasis case was reported in Hokkaido, the northernmost of the four main islands of Japan^25^. This patient had never lived in Okinawa or Kyushu and had no history of traveling abroad. The *Strongyloides* worms isolated from this patient were atypical in three ways: (1) These were the only worms in Japan to have Cox1 haplotype belonging to clade Ib (H010, Fig. 2); (2) Of the 521 isolates we analyzed in the present study, only one worm from this Hokkaido patient possessed the heterozygous genotype for 18S rDNA 458T/A polymorphism (Table 2); (3) Of all the Japanese human isolates, one worm from this patient carried the 4T/5T genotype. The source of the *Strongyloides* infection in this patient is unclear, and we believe that further epidemiological study is needed.

The evolutionary history of *S*. *stercoralis* has not been extensively studied. Having *S*. *procyonis* (parasites of raccoons) as a closest extant relative to *S*. *stercoralis* may indicate that both species share a common ancestor, that was most likely be a parasite of ancestral Caniformia. In this case, it might be assumed that *S*. *stercoralis* evolved as a parasite of canids originally, and later spread into humans as a result of domestication of *Canis lupus*. Our epidemiological data suggest that *S*. *stercoralis* type B identified in the present study have not adapted to infect humans, and this might be the ancestral phenotype of this species. This hypothesis is most likely and can be further assessed by analysis of more *Strongyloides* samples from humans, dogs, and dog-like (Caniformia) animals from diverse geographical locations using genome-wide sequence information^26^.

## Methods

### Ethics statement

The procedures used in this study were approved by the ethical committees of the University of Medicine 1, Yangon, Myanmar; the Prince of Songkla University, Thailand; and the University of Ryukyus, Japan. All work was performed in accordance with the relevant guidelines and regulations. Written informed consent was obtained from all volunteers who provided a stool specimen. In some cases, nematodes were isolated during routine fecal examinations at clinical laboratories (indicated as “clinical case” in Table 8). In such cases, no informed consent was sought for the use of parasite material. The volunteers and cases identified to be infected with *S*. *stercoralis* were treated with anthelminthic drugs. For collection of dog feces from streets or floors of animal shelters, no ethics committee approval or waiver of ethics approval was sought, in line with local policy.

**Table 8:** Summary of *Strongyloides* isolates used in this study.

Host	Country	Code	Sampling type	Sampling location	# of hosts (feces)	# of *Strongyloides* isolates	Reference	GenBank accession (Cox1 sequences)
Human	Japan	HKD	Clinical case	Hokkaido	1	3	This study	LC179026–LC179028
		KGS	Clinical case	Kagoshima	1	7	This study	LC179148–LC179154
		MYZ	Clinical case	Miyazaki	1	5	This study	LC179231–LC179235
		RKY	Care facility survey	Okinawa	6	34	This study	LC179360–LC179393
		YKS	Clinical case	Kagoshima	1	8	This study	LC179535–LC179542
		JPH	Clinical cases	Okinawa, Shizuoka, Tokyo	4	4	Hasegawa *et al*.^10^	AB526298–AB526301
	Myanmar	HTB	Field survey	Htantabin	24	119	This study	LC179029–LC179147
		MGD	Hospital based survey	Yangon	13	60	This study	LC179166–LC179225
		TBN	Field survey	Thabaung	6	27	This study	LC179459–LC179485
		TLY	Field survey	Thanlyin	6	28	This study	LC179499–LC179526
	Thailand	PSU	Hospital based study	Hat Yai	16	106	This study	LC179254–LC179359
	Laos	KU	Field survey	Three different provinces	39	39	Laymanivong *et al*.^33^	KU962139–KU962148 KU962150–KU962178
	Uganda	UGL	Clinical case	Gulu	1	8	This study	LC179527–LC179534
	Tanzania	TZH	Clinical case	Mahale	1	1	Hasegawa *et al*.^10^	AB526297
	Central African Republic	CAR	Field survey	Dzanga	3	3	Hasegawa *et al*.^32^	LC085498–LC085500
Dog	Japan	KYT	Clinical case	Kyoto	1	11	This study	LC179155–LC179165
		MIE	Clinical case	Mie	1	5	This study	LC179226–LC179230
		NGN	Clinical case	Nagano	1	10	This study	LC179244–LC179253
		STM	Clinical case	Saitama	1	3	This study	LC179456–LC179458
		FUK	Clinical case	Fukuoka	1	3	This study	LC179023–LC179025
		JPD	Unknown	Hyogo, Kanagawa	2	2	Hasegawa *et al*.^10^	AB526302–AB526303
	Myanmar	BAS	Animal shelter survey	Bago	1	1	This study	LC179022
		NDA	Animal shelter survey	North Dagon	3	8	This study	LC179236–LC179243
		SCT	Street survey	Sanchaung	22	62	This study	LC179394–LC179455
		TLD	Street survey	Thanlyin	5	13	This study	LC179486–179498
Chimp	Tanzania	TZC	Field survey	Mahale	1	1	Hasegawa *et al*.^10^	AB526305

Multiple nematodes were collected from a single fecal specimen. Both the number of hosts and number of nematodes isolated are shown. In the case of Myanmar dogs, for the calculation of host animal numbers, we assumed that each fresh fecal sample found on the streets or floors of animal shelters belonged to different dogs. We also included Cox1 sequence data available from the NCBI Nucleotide database.

### Study areas

#### Samples from humans

**Figure 5:**
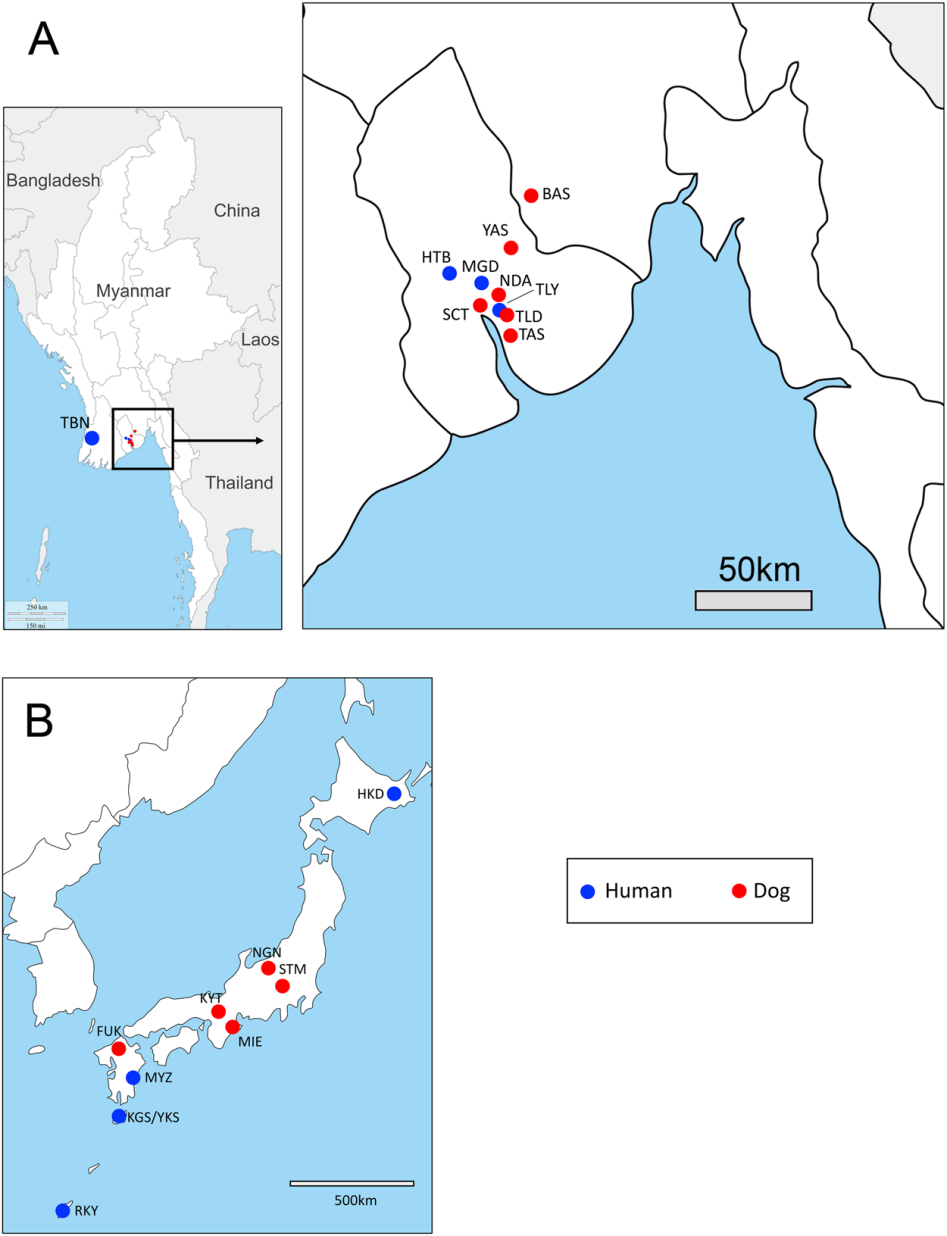
Map of Myanmar and Japan showing the geographical origin of the *Strongyloides* nematode samples. Maps were created with Adobe Creative Suite 5.5 and Microsoft PowerPoint version 15.22 from templates obtained from d-maps.com. (Myanmar: http://www.d-maps.com/carte.php?num_car=4162&lang=en, Japan: http://www.d-maps.com/carte.php?num_car=74&lang=en). Three-letter codes indicate sampling locations. Further details of each sampling location are summarized in Table 8.

In Myanmar, human fecal samples were obtained from healthy volunteers at three locations (Fig. 5A): Htantabin (Yangon Division, sample code: HTB), Thabaung (Ayeyawady Division, TBN), and Thanlyin (Yangon division, TLN). Additionally, feces samples were obtained from HIV-positive individuals at Mingaladon Specialist Hospital (MGD), also located in Yangon. In Thailand, human fecal samples were collected at the Prince of Songkla University hospital (PSU) from patients who were admitted owing to various medical conditions. In Japan, human fecal samples were obtained from volunteers in Okinawa prefecture (RYK). Additionally, fecal specimens from three clinically suspected strongyloidiasis cases were used in this study. One sample was from an individual from Yakusima (YKS), one was from a patient in Kagoshima (KGS), and the other was from a patient in Hokkaido (HKD). The sampling locations in Japan are shown in Fig. 5B. In Uganda, we obtained a sample from one clinically suspected strongyloidiasis case at St. Mary’s Hospital Lacor, Gulu.

#### Samples from dogs

We conducted two street surveys and two animal shelter surveys at areas inside or close to Yangon city (Yangon and Bago Divisions, Fig. 5A). Fresh dog feces were collected from the streets or the shelter cage floors. Fecal portions not in contact with the ground were collected in stool containers for transportation. To estimate the host number for dog feces sampling, we assumed that each feces found on street or on floor belonged to different dogs.

#### Sample processing

Parasitological examination was conducted using the agar plate technique^27^. Approximately 2 g of feces was placed on the center of 2% agar prepared in 60-mm diameter petri dishes. The plates were incubated for up to four days at room temperature and then examined under a dissection microscope for the presence of nematodes. The worm lysis solution was prepared by mixing 0.5 volumes of proteinase K (>600 mAU/mL solution) (Qiagen, Hilden, Germany), 0.5 volumes of 1 M dithiothreitol (DTT), and 10 volumes of DirectPCR Lysis Reagent (Tail) (Viagen Biotech, Inc., Los Angeles, CA)^23^. Nematodes found on agar plates were individually picked and put in PCR tubes containing 10 µL of the worm lysis solution. For lysis, the tubes were incubated at 60 °C for 20 minutes. They were then incubated at 95 °C for 20 minutes to inactivate proteinase K. The tubes containing worm lysate were stored at −30 °C until analyzed.

### PCR and DNA sequencing

A segment (935 bp) of the 18S rDNA gene corresponding to positions −2 to 933 of reference sequence AF279916 (GenBank) was amplified using primer pairs 988F (5′-ctcaaagattaagccatgc-3′) and 1912R (5′-tttacggtcagaactaggg-3′)^28^. The identification of the infecting nematode species was based on the nucleotide sequence of the 3′ region of this PCR product. The same primer, 1912R, was used for the sequencing reaction. A 333-bp region, which spans positions 315–683, was selected for species determination. This locus contains two hyper-variable regions, HVR-II (603–608) and HVR-III (631–651)^8^, which were reported to be useful for species specific identification among different *Strongyloides* species. If the 333-bp region, which corresponds to nucleotide positions 315–683 of *S*. *stercoralis* 18S rDNA reference sequence (AF279916), perfectly matched to the reference or matched with only one base difference at position 458 (known to be polymorphic among *S*. *stercoralis* ^11^), we considered the nematodes tested to be *S*. *stercoralis*. The HVR-I 4T/5T genotypes were determined by sequencing the 5′-region of this PCR product with the primer 988F. For samples in which sequencing traces could not be interpreted directly due to the heterozygosity at this locus, InDelligent v.1.2^29^ was used to computationally reconstruct the two allelic sequences.

A segment (1869 bp) of the mitochondrial genome^30^ (positions 5237–7106 of AJ558163; GenBank) including the entire open reading frame (ORF) of mitochondrial cytochrome c oxidase subunit 1 (Cox1) gene was amplified by PCR using the primer pairs ENM175 (5′-tttctggtgctcttcgactg-3′) and ENM176 (5′-aaaacaacacccataagactacaaaa-3′). The PCR products were purified in the same way as for the 18S rDNA gene. The PCR products were sequenced using ENM175 and ENM178 (5′-tggcttgttttgttgataatgg-3′). After trimming low-quality reads at both ends of the chromatogram, the sequences obtained with ENM175 and ENM178 were assembled into contigs. Erroneous base calls were corrected manually. These contigs were then aligned using ClustalX 2.1^31^. The aligned sequences were further trimmed to have a fixed length of 711 bp, which corresponds to positions 5656–6366 of AJ558163.

The Cox1 sequences that we obtained (521 sequences) and those from a database (50 sequences, NCBI database)^10, 32, 33^ were combined to form a single dataset.

A gDNA region that includes a portion of 28S rDNA was amplified using primer pairs ENM189 (5′-gtaaacgtaagtcattagcttacattg-3′) and ENM190 (5′-cgcactactatggctataactgc-3′). To sequence this PCR product, ENM190 and ENM270 (5′-ggacgtgaaaccgatacgat-3′) were used as sequencing primers. Erroneous base calls were corrected manually. The sequences obtained with both primers were assembled into contigs. The contigs were then aligned and trimmed so as to be aligned with the reference sequence (positions of 82–815 of U39489.1, GenBanK) The length of such trimmed contigs varied between 733 to 736 bp.

A gDNA region containing a putative MSP gene was amplified using the primer pair ENM246 (5′-caatatgatttagatattgctcaagc-3′) and ENM247 (5′-gtatttttacttacttcacctcc-3′), that were designed based on a reference gene sequence, SSTP_0000122000.1 (WormBase ParaSite; parasite.wormbase.org). The resultant PCR products (801 bp in length) were subjected to sequencing from both directions using the same primers used for the PCR. The sequences obtained with both primers were assembled into contigs. After manual error corrections, the contigs were aligned using ClustalX 2.1. The aligned contigs were trimmed into a fixed length of 551 base pairs that corresponds to positions 1,324,934–1,325,484 on scaffold 0000001 (*S*. *stercoralis* genome assembly v2.0.4)^26^.

In this study, we used the following rule for counting: after analyzing multiple worms isolated from a single host, if only a single genotype/haplotype was identified, the host was counted only once for this particular genotype/haplotype. However, when multiple genotypes/haplotypes were identified, the host was counted once for each genotype/haplotype. We also applied this rule to the haplotype network analysis (below).

### Phylogenetic and haplotype network analyses

A total of 571 Cox1 sequences were collapsed into haplotypes using DNACollapser^34^. The MEGA7 program^35^ was used to build a maximum-likelihood tree using Cox1 haplotypes obtained above based on the Tamura-Nei model.

In case of the 28S rDNA sequence, we acquired successful sequencing results from 311 out of 521 samples. The contig sequences from these 311 samples were collapsed into haplotypes. Using these haplotypes, a neighbor-joining phylogenetic tree was constructed using the MEGA7 program.

For analysis of the MSP gene, sequences from each individual nematode were used without collapsing into haplotypes sequences to construct a phylogenetic tree.

A median-joining network for Cox1 haplotypes was obtained and visualized using Network 5.0.0.0 and Network Publisher 2.1.1.2 (fluxus-engineering.com).

### Morphometrics of the *S*. *stercoralis* isolates belonging to Cox1 clade II

For morphometric analysis, an additional worm sampling was conducted. Free-living male and female adult worms were picked from an agar plate bearing a Myanmar dog fecal specimen and then heat-killed (1 minute at 60 °C). A part of the sample was fixed in 10% formalin for morphological analysis and the remainder was preserved in 75% ethanol for DNA sequencing. Images of each formalin-fixed nematode were captured using an Olympus BX51 microscope equipped with a DP71 digital camera. The measurements were carried out using Olympus cellSens imaging software.

### Accession codes

Sequencing data obtained in this study were submitted to DDBJ (DNA Data Bank of Japan) under accession numbers: Cox1: LC179022–LC179542, 28S rDNA: LC259531–LC259841, and MSP: LC184615, LC184617, LC184622, LC184624, LC184715, LC184717, LC184729, LC184748, LC184771, LC184777, LC184780, LC184811, LC184820, LC184821, LC184824, LC184829, LC184840, LC184844, LC184895, LC184920, LC184923, LC184924, LC184927, LC184930, LC184932, LC184935, LC184938, LC184940, LC184943, LC184944, LC184946.

## Electronic supplementary material

Supplementary Table S1

Supplementary Table S2
